# Long-term depression-inducing stimuli promote cleavage of the synaptic adhesion molecule NGL-3 through NMDA receptors, matrix metalloproteinases and presenilin/γ-secretase

**DOI:** 10.1098/rstb.2013.0158

**Published:** 2014-01-05

**Authors:** Hyejin Lee, Eun-Jae Lee, Yoo Sung Song, Eunjoon Kim

**Affiliations:** 1 Department of Biological Sciences, Korea Advanced Institute of Science and Technology (KAIST), Daejeon 305-701, Korea; 2 Center for Synaptic Brain Dysfunctions, Institute for Basic Science (IBS), Daejeon 305-701, Korea

**Keywords:** long-term depression, synaptic adhesion molecules, NMDA receptors, metalloproteinase, γ-secretase

## Abstract

Long-term depression (LTD) reduces the functional strength of excitatory synapses through mechanisms that include the removal of AMPA glutamate receptors from the postsynaptic membrane. LTD induction is also known to result in structural changes at excitatory synapses, including the shrinkage of dendritic spines. Synaptic adhesion molecules are thought to contribute to the development, function and plasticity of neuronal synapses largely through their trans-synaptic adhesions. However, little is known about how synaptic adhesion molecules are altered during LTD. We report here that NGL-3 (netrin-G ligand-3), a postsynaptic adhesion molecule that trans-synaptically interacts with the LAR family of receptor tyrosine phosphatases and intracellularly with the postsynaptic scaffolding protein PSD-95, undergoes a proteolytic cleavage process. NGL-3 cleavage is induced by NMDA treatment in cultured neurons and low-frequency stimulation in brain slices and requires the activities of NMDA glutamate receptors, matrix metalloproteinases (MMPs) and presenilin/γ-secretase. These results suggest that NGL-3 is a novel substrate of MMPs and γ-secretase and that NGL-3 cleavage may regulate synaptic adhesion during LTD.

## 1. Introduction

Synaptic adhesion molecules play important roles in the regulation of synaptic development, function and plasticity [1–14]. A large number of synaptic adhesion molecules have recently been identified. These include neuroligins, neurexins, SynCAMs (synaptic cell adhesion molecules), LRRTMs (leucine-rich repeat transmembrane neuronal proteins, NGLs (netrin-G ligands), SALMs (synaptic adhesion-like molecules), netrin-Gs, LAR-RPTPs (leucocyte common antigen-related protein-receptor-type protein tyrosine phosphatases), EphB receptors (EphB class ephrin receptors), GluRδ2 (δ2 glutamate receptor), Cbln1 (cerebellin 1 precursor protein), TrkC (tropomyosin receptor kinase C), Slitrks (SLIT and NTRK-like proteins), MDGAs (MAM domain-containing glycosylphosphatidylinositol anchor proteins), IL1RAPL1 (interleukin-1 receptor accessory protein-like 1) [15–28] and IL1RAcP (interleukin-1 receptor accessory protein) [29].

Synaptic adhesion molecules are thought to contribute to synaptic development largely through their trans-synaptic adhesions, but relatively little is known about how synaptic adhesions are structurally weakened and how this contributes to functional weakening of synapses. It has recently been shown that neuroligin-1 is cleaved in an activity-dependent manner through mechanisms requiring the activation of metalloproteinases MMP-9 (matrix metalloproteinase 9) and ADAM-10 (a disintegrin and metalloproteinase 10); this cleavage leads to both structural and functional weakening of synapses [30,31]. However, it remains unclear whether other synaptic adhesion molecules are similarly regulated. It is also not certain whether long-term depression (LTD), which is accompanied by the shrinkage of dendritic spines, loss of F-actin in spines and separation of pre- and postsynaptic structures [32–34], leads to proteolytic cleavage of synaptic adhesion molecules.

NGLs (netrin-G ligands) are a family of postsynaptic adhesion molecules with three known members: NGL-1, NGL-2 and NGL-3 [9]. The C-terminal tails of NGLs interact with the postsynaptic scaffolding protein PSD-95 [19], suggesting that these interactions promote the recruitment of PSD-95-associated receptors and signalling molecules to the sites of presynaptic release [35]. Extracellular domains of NGL-1 and NGL-2 interact with netrin-G1 and netrin-G2, respectively [18,19]; these latter molecules are axonally enriched glycosylphosphatidyl inositol (GPI)-anchored adhesion proteins [36–39]. NGL-3 interacts with members of the LAR family of receptor tyrosine phosphatases (LAR, PTPδ and PTPσ) [20,21,40], which are critically involved in regulating presynaptic development and function [41]. More recently, LAR family proteins have been shown to interact with diverse postsynaptic adhesion molecules, including TrkC, Slitrks, IL1RAPL1 and IL1RAcP [20–22,25–29] and have emerged as novel organizers of presynaptic development, although a postsynaptic role in synapse development and maintenance has also been suggested [42].

Matrix metalloproteinases (MMPs) are a group of zinc-dependent endopeptidases known to process extracellular matrix components and cell surface proteins. In the nervous system, MMPs are involved in various brain functions and dysfunctions, including brain development, synaptogenesis, synaptic plasticity, learning and memory, neuron–glia interactions, neuronal injury and neurological and neuropsychiatric disorders [43–54]. Of the numerous MMPs expressed in the nervous system, MMP-9 has been extensively characterized and shown to regulate late-phase long-term potentiation (LTP), dendritic spine morphology and learning and memory, through mechanisms including integrin signalling and NMDA (*N*-methyl-d-aspartate) receptor trafficking and function [55–65]. MMP-9, however, does not regulate early-phase LTP, presynaptic release, NMDA receptor-dependent LTD or metabotropic glutamate receptor (mGluR)-dependent LTD. Although a study using general MMP inhibitors has suggested a role for MMPs in the regulation of LTD [66], the regulation of LTD by MMPs, unlike MMP-dependent regulation of LTP, is not well understood. In addition, it remains unclear which substrate proteins mediate MMP-dependent regulation of LTP and LTD, although a recent study has identified subunits of NMDA receptors, which directly regulate synaptic plasticity [67], as novel substrates of MMP-7 [68]. Notably, many substrates of MMPs, including cadherins, ephrins, Eph receptors, β-dystroglycan and neuroligin-1, are SynCAMs [30,31,69–76]. Given the increasing recognition that synaptic adhesion molecules are involved in the regulation of synaptic plasticity [77–81], it is possible that MMP-dependent cleavage of synaptic adhesion molecules could contribute to regulation of synaptic plasticity.

The presenilin/γ-secretase complex is a group of membrane-embedded proteinases that is composed of four proteins, including presenilin, nicastrin, Aph-1 and Pen-2 [82]. Well-known substrates of γ-secretase include the amyloid β-protein precursor (APP) and Notch, which have been implicated in Alzheimer's disease and brain development, respectively. A large number of γ-secretase substrates have recently been identified and the number is now approaching approximately 90 [83–85]. Notably, many γ-secretase substrates are synaptic surface proteins [83–85], including N-cadherin [86], ErbB4 [87,88], nectin-1α [89], syndecan-1/2/3 [85,90], GluR3 (GluA3) [91], ephrinB1/2 [92–94], EphA4/B2 [95,96], LAR [97], neurexin-1/3β [98,99] and neuroligin-1 [30,31]. This suggests that γ-secretase could act on these proteins to regulate synapse structure and function.

In this study, we tested whether NGL-3, used as a model adhesion molecule, undergoes a series of proteolytic cleavages in a neuronal activity-dependent manner. We found that LTD-inducing chemical and electrical stimuli caused proteolytic cleavage of NGL-3 in a manner requiring the activation of NMDA receptors, MMPs and γ-secretase. These results suggest that NGL-3 cleavage may regulate synaptic structure and function during LTD.

## 2. Results

### (a) NMDA treatment of cultured neurons induces NGL-3 cleavage

**Figure 1.**
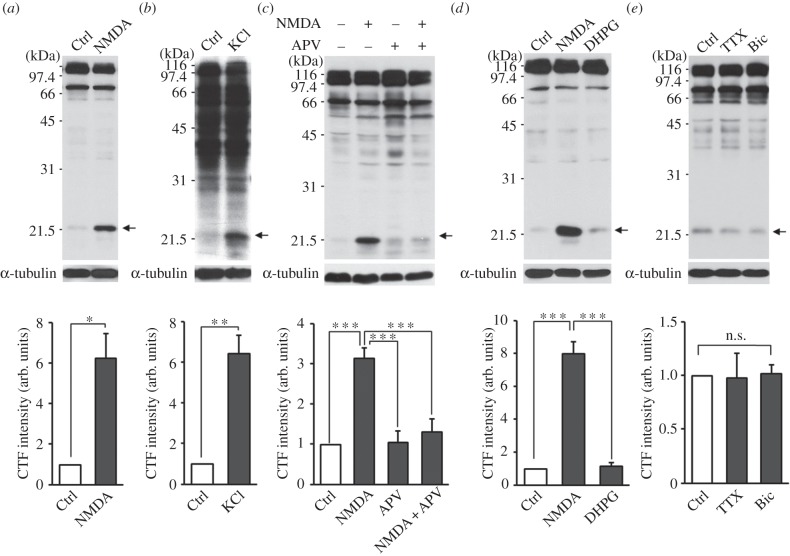
NGL-3 cleavage requires NMDAR activation, but not mGluR activation or chronic changes in neuronal activity. (*a*) NMDA treatment of cultured neurons induces NGL-3 cleavage. Rat hippocampal neurons at days *in vitro* (DIV) 18–21 were stimutated with NMDA (20 µM, 3 min), followed by direct lysis of the neurons in SDS-PAGE sample buffer and immunoblotting with NGL-3 antibodies (#1948). For quantitative analyses, the intensity of the 22 kDa band (indicated by an arrow) was normalized to that of α-tubulin and compared with other normalized intensities. The bar graphs represent mean ± s.e.m.; *n* = 3, *p < 0.05, Student's *t*-test. (*b*) KCl-dependent depolarization induces NGL-3 cleavage. Rat hippocampal neurons at DIV 18–21 were stimutated with KCl (50 mM, 5 min) followed by immunoblotting; *n* = 4, **p < 0.01, Student's *t*-test. (*c*) NMDA receptor activation is required for NMDA-induced NGL-3 cleavage. Rat hippocampal neurons pretreated with APV (NMDA receptor antagonist; 50 µM, 30 min) were stimulated with NMDA (20 µM, 3 min) and analysed by immunoblotting; *n* = 5, ***p < 0.001, one-way ANOVA. (*d*) Activation of NMDA receptors, but not mGluRs, leads to NGL-3 cleavage. Rat hippocampal neurons at DIV 18–21 were stimutated with NMDA (20 µM, 3 min) or DHPG (group I mGluR agonist; 50 µM, 30 min); *n* = 3, ***p < 0.001, one-way ANOVA. (*e*) Chronic inhibition or activation of cultured neurons does not induce NGL-3 cleavage. Rat hippocampal neurons at DIV 18–21 were stimutated with tetrodotoxin (TTX; 1 µM for 48 h) or bicuculline (Bic; 10 µM for 36 h); *n* = 3, n.s., not significant, one-way ANOVA. arb. units, arbitrary units; Ctrl, control.

To test whether LTD induction leads to the cleavage of NGL-3, we first treated cultured hippocampal neurons with NMDA (20 μM, 3 min), which is known to induce chemical LTD in brain slices [100] and a long-lasting decrease in surface levels of AMPA receptors in cultured neurons [101,102]. Immunoblot analyses showed that NMDA treatment of cultured neurons increased the levels of an NGL-3 fragment (approx. 22 kDa) recognized by an NGL-3 antibody raised against the cytoplasmic region of NGL-3 (amino acids 622–690; #1948) (figure 1*a*). Three other antibodies raised against the cytoplasmic region of NGL-3 recognized the same 22 kDa band (see electronic supplementary material, figure S1), suggesting the authenticity of the band as NGL-3 C-terminal fragments (hereafter termed NGL-3-CTFs). The small levels of NGL-3-CTFs before NMDA treatment suggest that NGL-3 cleavage occurs under basal conditions. A faint band beneath the NGL-3-CTF band is not likely to be an alternative or subsequent cleavage product, because it is not recognized by other NGL-3 antibodies (see electronic supplementary material, figure S1).

l-Glutamate treatment (50 μM, 1 min) of cultured hippocampal neurons, which has been used to induce internalization of AMPA receptors in cultured neurons [103], induced the same NGL-3 cleavage (see electronic supplementary material, figure S2*a*), similar to the results of the NMDA treatment. In addition, KCl treatment (50 mM, 5 min) induced NGL-3 cleavage (figure 1*b*), suggesting that general depolarization of neurons can induce NGL-3 cleavage.

### (b) NGL-3 cleavage requires NMDA receptor activity but not mGluR activity or chronic changes in neuronal activity

NMDA-induced cleavage of NGL-3 may involve the activation of NMDA receptors. Indeed, incubation of cultured neurons with APV (d-2-amino-5-phosphovalerate, 50 μM), an antagonist of NMDA receptors, 30 min before and during NMDA treatment (20 μM, 3 min) blocked NGL-3 cleavage (figure 1*c*). In addition, APV also inhibited glutamate- and KCl-induced NGL-3 cleavage (see electronic supplementary material, figure S2*a*,*b*).

By contrast, incubation of cultured neurons with DHPG ((*RS*)-3,5-dihydroxyphenylglycine, 50 μM, 30 min), an agonist of group I mGluRs (mGluR1/5) known to induce mGluR-dependent LTD (mGluR-LTD) in brain slices [104–107] and AMPA receptor internalization in cultured neurons [108], had no effect on NGL-3 cleavage, suggesting that mGluR activation does not induce NGL-3 cleavage (figure 1*d*). In addition, blocking neuronal network activity with tetrodotoxin (1 μM, 48 h) or enhancing network activity with bicuculline (10 μM, 36 h) had no effect on NGL-3 cleavage (figure 1*e*). These results suggest that neither mGluR activation nor chronic modulation of neuronal activity induces NGL-3 cleavage.

### (c) Long-term depression-inducing low-frequency stimulation causes NGL-3 cleavage in brain slices

**Figure 2.**
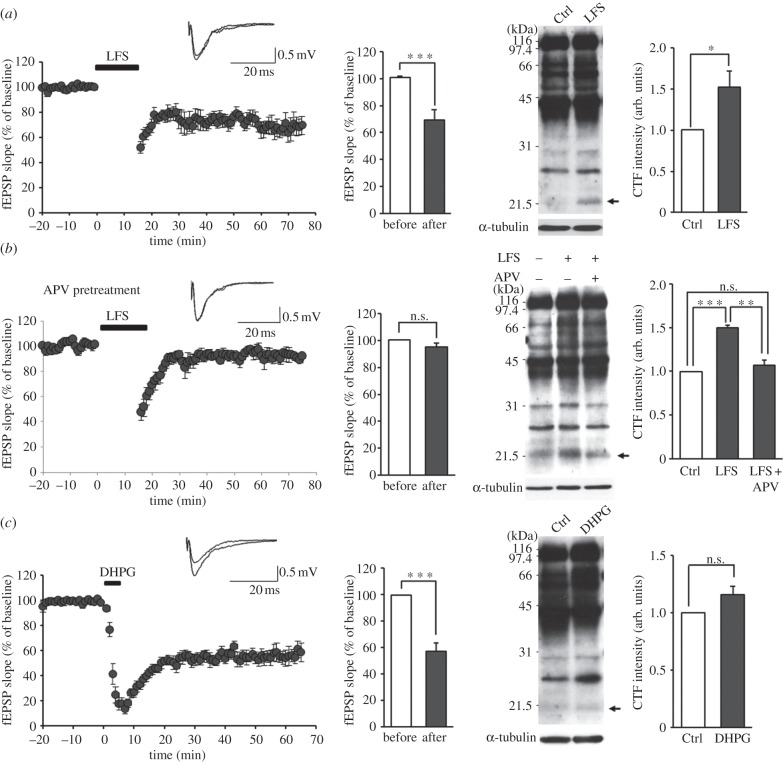
LTD-inducing low-frequency stimulation induces NGL-3 cleavage in hippocampal slices. (*a*) Low-frequency stimulation in slices induces NGL-3 cleavage. The Schaffer collateral pathway of acute mouse hippocampal slices (3–4 weeks old) was stimulated by low-frequency stimuli (LFS, 1 Hz, 15 min), followed by immunoblot analysis of hippocampal lysates using NGL-3 antibodies (#2020). The induction of LFS-LTD was confirmed by comparing the average slope of fEPSPs (field excitatory postsynaptic potentials) before stimulation with that during the last 5 min of recording. The top panel shows sample EPSPs. The bar graphs represent mean ± s.e.m; *n* = 4 slices from three mice, *p < 0.05, ***p < 0.001, Student's *t*-test. (*b*) NMDA receptor activation is required for LFS-induced cleavage of NGL-3-CTF. Acute mouse hippocampal slices (3–4 weeks old) were incubated with APV (NMDA receptor antagonist; 50 µM) throughout the experiment starting from 20 min before LFS, followed by immunoblot analysis of hippocampal lysates. The blockade of LFS-LTD was confirmed by comparing the average slope of fEPSPs before stimulation with that during the last 5 min of recording; *n* = 3 slices from two mice, **p < 0.01, ***p < 0.001, n.s., not significant, Student's *t*-test and one-way ANOVA. (*c*) DHPG treatment in slices does not induce NGL-3 cleavage. Acute mouse hippocampal slices (3–4 weeks old) were stimulated with DHPG (50 µM, 5 min), followed by immunoblot analysis of hippocampal lysates. The induction of mGluR-LTD by DHPG was confirmed by comparing the average slope of fEPSPs before stimulation with that during the last 5 min of recording. The top panel shows sample EPSPs; *n* = 6 slices from four mice, ***p < 0.001, n.s., not significant, Student's *t*-test. Ctrl, unstimulated control slices.

We next tested whether LTD-inducing electrical stimulation in brain slices causes NGL-3 cleavage. To accomplish this, we stimulated the Schaffer collateral pathway in mouse hippocampal slices (three weeks) by low-frequency stimulation (LFS, 1 Hz, 900 pulses), a stimulation paradigm known to cause NMDA receptor-dependent LTD in rat and mouse hippocampal slices [109,110], followed by confirmation of LTD induction by electrophysiological measurements, and immunoblotting analysis of hippocampal lysates. We found that LFS-LTD induced a significant increase in NGL-3 cleavage (figure 2*a*), similar to the results of chemical LTD induction in cultured neurons (figure 1). The presence of NGL-3-CTFs in hippocampal slices before LFS-LTD indicates that NGL-3 cleavage occurs under basal conditions, similar to the results from cultured neurons. Incubation of brain slices with APV (50 μM) blocked LFS-induced NGL-3 cleavage (figure 2*b*), suggesting that NMDA receptor activation is required. In contrast to LFS-LTD, inducing mGluR-LTD by incubation of hippocampal slices with DHPG (50 μM, 5 min) had no effect on NGL-3 cleavage (figure 2*c*), similar to the results of DHPG stimulation in cultured neurons (figure 1*d*). A similar NGL-3 cleavage could also be observed in rat slices stimulated by LFS (data not shown).

### (d) NGL-3 cleavage requires matrix metalloproteinase activity

**Figure 3.**
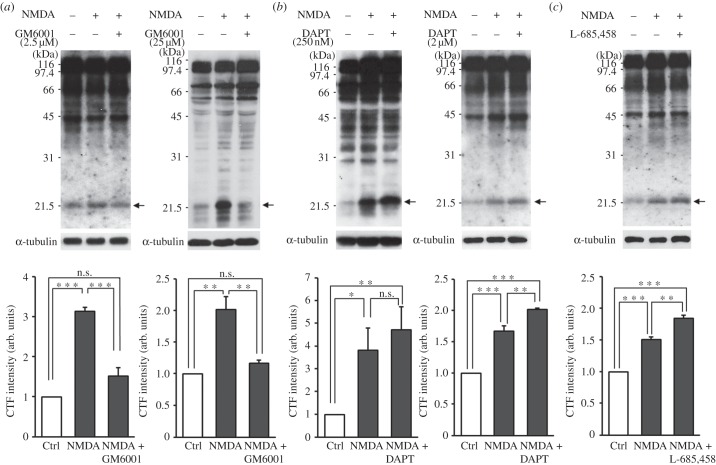
NGL-3 cleavage is blocked by inhibition of MMPs and presenilin/γ-secretase. (*a*) MMP inhibition blocks NMDA-induced NGL-3 cleavage. Rat hippocampal neurons at DIV 18–21 were incubated with GM6001 (2.5 and 25 µM, 30 min) before and during NMDA stimulation (20 µM, 3 min), followed by immunoblotting. The bar graphs represent mean ± s.e.m; *n* = 4; **p < 0.01, ***p < 0.001, n.s., not significant, one-way ANOVA. (*b*) γ-Secretase inhibition by DAPT blocks NGL-3 cleavage, leading to an increase in the levels of NGL-3-CTFs. Hippocampal neurons pretreated with the γ-secretase inhibitor DAPT (250 nM, 2 h ; 2 µM, 3 h) were stimulated with NMDA (20 µM, 3 min); *n* = 5, *p < 0.05, **p < 0.01, ***p < 0.001, n.s., not significant, one-way ANOVA. (*c*) γ-Secretase inhibition by L-685,458 blocks NGL-3 cleavage, leading to an increase in the levels of NGL-3-CTFs. Hippocampal neurons pretreated with the γ-secretase inhibitor L-685,458 (1 µM, 30 min) were stimulated with NMDA (20 µM, 3 min); *n* = 3, **p < 0.01, ***p < 0.001, one-way ANOVA.

NMDA receptor activation often leads to the activation of metalloproteinases [30,31,111]. We thus tested whether NMDA-induced NGL-3 cleavage requires MMP activity. Inhibition of MMPs with two different concentrations (2.5 and 25 μM) of GM6001, a broad-spectrum MMP inhibitor [112,113], for 30 min before and during NMDA treatment blocked NGL-3 cleavage in cultured neurons (figure 3*a*), suggesting that NGL-3 cleavage induced by NMDA receptor activation requires activation of MMPs.

### (e) NGL-3 cleavage requires γ-secretase activity

MMP cleavage of surface membrane proteins is usually followed by subsequent cleavage of their CTFs by γ-secretase [82]. To test whether this also occurs for NGL-3, we treated cultured hippocampal neurons with DAPT (*N*-[*N*-(3,5-difluorophenacetyl)-l-alanyl]-*S*-phenylglycine *t*-butyl ester), a γ-secretase inhibitor, at two different concentrations (250 nM and 2 μM) for 2–3 h before and during NMDA treatment. The levels of NGL-3-CTFs in cultured neurons induced by NMDA treatment combined with DAPT inhibition (2 μM) were higher than those induced by NMDA alone (figure 3*b*). We obtained a similar result by using L-685,458 (1 μM), another γ-secretase inhibitor (figure 3*c*). These results suggest that NGL-3-CTFs can be further cleaved by γ-secretase.

## 3. Discussion

Our data indicate that LTD-inducing stimuli promote proteolytic cleavage of NGL-3 in a manner that requires the activation of NMDA receptors, MMPs and presenilin/γ-secretase. These results suggest that (i) NGL-3 is a novel substrate of MMPs and γ-secretase, (ii) LTD induction promotes NGL-3 processing and (iii) NGL-3 may regulate excitatory synapse structure and function during LTD.

**Figure 4.**
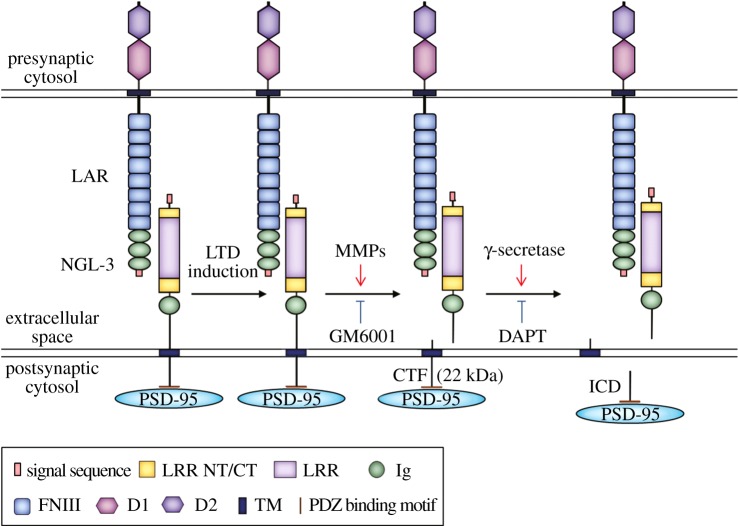
A schematic diagram showing that NGL-3 cleavage at the postsynaptic surface occurs in a manner that requires the activation of NMDA receptors, MMPs and γ-secretase, and may involve the destabilization of presynaptic and cytoplasmic binding partners of NGL-3. Cleavage of full-length NGL-3 is induced by LTD-inducing stimuli. MMPs (inhibited by the broad-spectrum MMP inhibitor GM6001) are thought to mediate the initial LTD-induced NGL-3 cleavage and generation of NGL-3-CTFs (22 kDa). γ-Secretase (inhibited by DAPT) may further cleave NGL-3-CTFs to generate NGL-3-ICDs, although they were not detectable in our immunoblots. Presynaptic LAR family receptor tyrosine phosphatases, which trans-synaptically interact with NGL-3, may undergo removal from the presynaptic site. Cytoplasmically, the postsynaptic scaffolding protein PSD-95, which binds the cytoplasmic tail of NGL-3, may lose its connection with NGL-3, contributing to destabilization of the synapse during LTD. LRRNT, leucine-rich repeat N-terminal domain; LRRCT, leucine-rich repeat C-terminal domain; LRR, leucine-rich repeats; Ig, immunoglobulin domain; TM, transmemrbane domain; PDZ binding motif, PDZ domain-binding motif; FNIII, fibronectin type III domain; D1 and D2, tyrosine phosphatase domains.

What might be the consequences of NGL-3 cleavage by MMPs and γ-secretase? One straightforward possibility would be the weakening of NGL-3-mediated trans-synaptic adhesion at excitatory synapses that undergo LTD (figure 4). In addition, γ-secretase-mediated cleavage of NGL-3-CTFs, the step following MMP cleavage, would further remove the C-terminal tail from NGL-3, destabilizing the interaction between NGL-3 and PSD-95 (figure 4).

NGL-3 trans-synaptically interacts with LAR family receptor protein tyrosine phosphatases (LAR, PTPδ and PTPσ) [20,21]. Therefore, NGL-3 cleavage may cause the removal of LARs from synapses, similar to the removal of presynaptic neurexins observed at the site of neuroligin-1 cleavage [30]. Functionally, LAR is a well-known regulator of presynaptic development and function [41,114–116]. Synaptic removal of LAR induced by NGL-3 cleavage is thus likely to exert a significant influence on presynaptic structure and function. Notably, LAR can be processed by ADAM-17/TACE (an α-secretase) and γ-secretase [97,117,118]. It is conceivable that NGL-3 and LAR may undergo simultaneous proteolytic cleavages by specific MMPs/ADAMs during LTD, although we could not test LTD-induced LAR cleavage in this study owing to a lack of suitable LAR antibodies.

Our results are reminiscent of the recently reported activity-dependent cleavage of neuroligin-1 mediated by MMP-9/ADAM-10 and γ-secretase [30,31]. Neurexin-1β and neurexin-3β, which interact with neuroligins, have also been shown to be substrates of γ-secretase [98,99]. The complex of neuroligins and neurexins has been clearly demonstrated to regulate diverse aspects of synapse development and function [2–4,13]. Therefore, these previous observations taken together with the results of this study support the notion that the cleavage of synaptic adhesion molecules by MMPs/ADAMs and γ-secretase regulates diverse aspects of synapse structure and function. One rather unique finding of the current study is that LTD-inducing low-frequency stimulation in slices leads to NGL-3 cleavage. It thus may be worth testing whether neuroligin-1 undergoes a similar LTD-induced cleavage and whether neuroligin-1 and NGL-3 act in a synergistic manner during LTD.

NGL-3 cleavage induced by LTD-inducing stimuli requires MMP activation. Because MMPs have been implicated in the regulation of synaptic plasticity including LTP and LTD [55–62,66], our data suggest that NGL-3 may be one of the downstream effectors that mediate MMP-dependent regulation of LTD. With regard to specific MMPs that may mediate NGL-3 cleavage, MMP-9 is unlikely to be involved because LTD is unaffected in MMP-9-deficient mice [58] and in slices with specific inhibition of MMP-9 [66]. Other possible MMPs include MMP-3 and MMP-7, which have been shown to regulate NMDA receptor function, synaptic plasticity, dendritic spine morphology, and learning and memory [62,68,119–126]. GM6001 used in this study is a broad-spectrum inhibitor that acts on both MMPs and ADAMs [112,113]. Therefore, ADAMs may also act on NGL-3, similar to the cleavage of neuroligin-1 by both MMP-9 and ADAM-10 [30,31]. However, ADAM-17 (also known as TACE) is unlikely to process NGL-3 because it is known to mainly regulate mGluR1/5-dependent LTD [127], and DHPG stimulation in cultured neurons or mGluR-LTD in brain slices does not induce NGL-3 cleavage (figures 1*d* and 2*c*).

NGL-3 cleavage requires γ-secretase activity. γ-Secretase has been shown to enhance LTD through the production of amyloid-β and endocytosis of NMDA receptors [128–130]. It is thus possible that NGL-3 also contributes to γ-secretase-dependent regulation of LTD. In addition, γ-secretase acts on synaptic adhesion molecules, including neurexins, neuroligins, cadherins, nectins, syndecans, LARs, ephrins and Eph receptors [83–85], and many of these molecules regulate synaptic plasticity [77,78,131–136]. Thus, the results of our study, taken together with these previous observations, corroborate the notion that γ-secretase regulates synaptic adhesion and plasticity through activity-dependent cleavage of synaptic adhesion molecules.

γ-Secretase action is usually preceded by the MMP-mediated cleavage of membrane proteins into N- and C-terminal fragments (NTFs and CTFs) [82–85]. CTFs are further processed by γ-secretase to generate intracellular domains (ICDs), which are known to regulate intracellular signalling in the cytoplasm or nucleus by, for instance, interacting with transcriptional regulators. Alternatively, ICDs are degraded by the proteasome for protein turnover. In this study, we could not observe detectable levels of NGL-3-ICDs. This suggests that NGL-3-ICDs may be labile and degraded by the proteasome, similar to the case of ICDs from neuroligin-1 [31]. However, this does not exclude the possibility that NGL-3-ICDs have some cytoplasmic functions. Notably, ICDs derived from NGL-3-interacting LAR translocate to the nucleus and regulate β-catenin-dependent gene expression [97].

A member of the LAR family, PTPδ (encoded by the *PTPRD* gene), has been associated with attention deficit/hyperactivity disorder (ADHD) [137] and restless leg syndrome [138], a disorder often comorbid with ADHD [139], autism spectrum disorder [140] and bipolar disorder [141]. This suggests the possibility that abnormalities in the trans-synaptic interactions between LAR family proteins and their postsynaptic partners including NGL-3 may contribute to the development of these disorders.

In summary, our data suggest that induction of LTD in neurons leads to proteolytic cleavage of NGL-3 in a manner requiring the activation of NMDA receptors, MMPs and γ-secretase. This cleavage may lead to the weakening of NGL-3-dependent trans-synaptic adhesion at excitatory synapses and contribute to structural and functional weakening of excitatory synapses during LTD.

## 4. Material and methods

### (a) Antibodies and animals

Guinea pig polyclonal NGL-3 antibodies (#2020 and #2021) were raised in this study using synthetic peptides mimicking the last 30 amino acid residues of NGL-3 (CGAKGPGLNSIHEPLLFKSCGSKENVQETQI). Rabbit polyclonal pan-NGL (#1583; against last 15 amino acid residues of NGL-2; CIIQTHTKDKVQETQI) [17] and rabbit polyclonal NGL-3 antibodies (#1948; against GST-NGL-3 amino acids 622–690) [21] have been described. α-Tubulin antibody was purchased from Sigma. Experiments on animals were performed in accordance with the guidelines of the Animal Welfare Committee of KAIST, Korea.

### (b) Quantification of immunoblot results

NGL-3-CTF bands were quantified by normalizing the integrated intensities of the 22 kDa bands to those of tubulin bands, and comparing these normalized values from treated cultured neurons or stimulated brain slices with those from untreated control neurons or slices.

### (c) Primary rat hippocampal neuron culture and drug treatment

Cultured hippocampal neurons were prepared from embryonic day 18 Sprague-Dawley rat brains. Dissociated neurons on poly-l-lysine coated (1 mg ml^−1^) coverslips were placed in neurobasal medium supplemented with B27 (Invitrogen), 0.5 mM l-glutamate and penicillin–streptomycin. For activation, cultured neurons at days *in vitro* (DIV) 18–21 were treated with NMDA (Sigma; 20 µM, 3 min), KCl (Sigma; 50 mM, 5 min) or l-glutamate (Sigma; 50 µM, 30 min). For induction of NMDA receptor-dependent LTD or mGluR-LTD, cultured neurons were treated with NMDA (20 µM, 3 min) and DHPG (Tocris; 50 μM, 30 min), respectively, and returned to normal conditioning medium. For the blockade of NMDA receptors, cultured neurons were pretreated with APV (Tocris; d-2-amino-5-phosphovalerate; 50 µM, 30 min), followed by stimulation with NMDA (20 µM, 3 min) in the presence of APV or with l-glutamate (50 µM, 1 min) or KCl (30 mM, 1 min) in the absence of APV. For chronic neuronal inhibition or activation, neurons were treated with tetrodotoxin (Tocris; TTX; 1 µM, 48 h) or with bicuculline (Tocris; Bic; 10 µM, 36 h). For blockade of metalloproteinase and γ-secretase activity, neurons were pretreated with GM6001 (Enzo Life Sciences; 2.5 and 25 µM, 30 min), DAPT (Sigma; 250 nM, 2 h; 2 µM, 3 h) and L-685,458 (Calbiochem; 1 µM, 30 min) before and during NMDA stimulation (20 µM, 3 min).

### (d) Electrophysiology

C57BL/6 background wild-type mice of both sexes at the age of postnatal day 21–28 were used for electrophysiological experiments. Sagittal hippocampal slices (400 μm thick) were prepared using a vibratome (Leica VT1200) in ice-cold dissection buffer containing (in mM) 212 sucrose, 25 NaHCO_3_, 5 KCl, 1.25 NaH_2_PO_4_, 0.5 CaCl_2_, 3.5 MgSO_4_, 10 d-glucose, 1.25 l-ascorbic acid and 2 Na-pyruvate bubbled with 95% O_2_/5% CO_2_. The slices were recovered at 32°C for 1 h in normal artificial cerebrospinal fluid (ACSF) (in mM: 125 NaCl, 25 NaHCO_3_, 2.5 KCl, 1.25 NaH_2_PO_4_, 2.5 CaCl_2_, 1.3 MgCl_2_, 10 d-glucose) and thereafter maintained at room temperature. Extracellular field recordings were performed to monitor LTD induction. Stimulation and recording pipettes were pulled from borosilicate glass capillaries (Harvard Apparatus) using a micropipette electrode puller (Narishege). Field excitatory postsynaptic potentials (fEPSPs) were recorded in the stratum radiatum of the hippocampal CA1 using pipettes filled with ACSF (1 M*Ω*). The Schaffer collateral pathway was stimulated every 20 s with pipettes filled with ACSF (0.3–0.5 M*Ω*). The stimulation intensity was adjusted to yield a half-maximal response, and three successive responses were averaged and expressed relative to the normalized baseline. After a stable baseline was established, NMDA receptor-dependent LTD and mGluR-LTD were induced by low-frequency stimulation (1 Hz, 15 min) and applying DHPG (50 µM, 5 min), respectively. To inhibit NMDA receptor-dependent LTD, APV (50 µM) was bath-applied throughout the recordings starting from 20 min before LFS, as reported previously [110]. Recordings were made by using MultiClamp 700B amplifier (Molecular Devices) under visual control with differential interference contrast illumination in an upright microscope (BX50WI; Olympus). Data were acquired by Clampex 10.2 (Molecular Devices) and analysed by Clampfit 10 (Molecular Devices). Picrotoxin (100 µM) was added during both LTD experiments. Immediately after electrophysiological recordings (60 min after LFS), mouse brain slices were homogenized in buffered sucrose (0.32 M sucrose, 4 mM HEPES, 1 mM MgCl_2_, 0.5 mM CaCl_2_, pH 7.3) with freshly added protease inhibitors for immunoblot analyses.

## Funding statement

This study was supported by the Institute for Basic Science (IBS).
